# Green leaf volatiles and jasmonic acid enhance susceptibility to anthracnose diseases caused by *Colletotrichum graminicola* in maize

**DOI:** 10.1111/mpp.12924

**Published:** 2020-02-27

**Authors:** Zachary Gorman, Shawn A. Christensen, Yuanxin Yan, Yongming He, Eli Borrego, Michael V. Kolomiets

**Affiliations:** ^1^ Department of Plant Pathology and Microbiology Texas A&M University College Station TX USA; ^2^ Department of Agriculture–Agricultural Research Service (USDA–ARS), Chemistry Research Unit Center for Medical, Agricultural, and Veterinary Entomology Gainesville FL USA; ^3^ State Key Laboratory of Crop Genetics and Germplasm Enhancement Nanjing Agricultural University Nanjing China; ^4^ Jiangxi Key Laboratory of Crop Physiology, Ecology, and Genetic Breeding Jiangxi Agricultural University Nanchang China; ^5^ Thomas H. Gosnell School of Life Sciences Rochester Institute of Technology Rochester NY USA

**Keywords:** *Colletotrichum graminicola*, green leaf volatile (GLV), hormone cross‐talk, jasmonic acid (JA), lipoxygenase (LOX), salicylic acid (SA), *Zea mays* (maize)

## Abstract

*Colletotrichum graminicola* is a hemibiotrophic fungus that causes anthracnose leaf blight (ALB) and anthracnose stalk rot (ASR) in maize. Despite substantial economic losses caused by these diseases, the defence mechanisms against this pathogen remain poorly understood. Several hormones are suggested to aid in defence against *C. graminicola*, such as jasmonic acid (JA) and salicylic acid (SA), but supporting genetic evidence was not reported. Green leaf volatiles (GLVs) are a group of well‐characterized volatiles that induce JA biosynthesis in maize and are known to function in defence against necrotrophic pathogens. Information regarding the role of GLVs and JA in interactions with (hemi)biotrophic pathogens remains limited. To functionally elucidate GLVs and JA in defence against a hemibiotrophic pathogen, we tested GLV‐ and JA‐deficient mutants, *lox10* and *opr7 opr8*, respectively, for resistance to ASR and ALB and profiled jasmonates and SA in their stalks and leaves throughout infection. Both mutants were resistant and generally displayed elevated levels of SA and low amounts of jasmonates, especially at early stages of infection. Pretreatment with GLVs restored susceptibility of *lox10* mutants, but not *opr7 opr8* mutants, which coincided with complete rescue of JA levels. Exogenous methyl jasmonate restored susceptibility in both mutants when applied before inoculation, whereas methyl salicylate did not induce further resistance in either of the mutants, but did induce mutant‐like resistance in the wild type. Collectively, this study reveals that GLVs and JA contribute to maize susceptibility to *C. graminicola* due to suppression of SA‐related defences.

## Graphical abstract

Jasmonic acid and green leaf volatiles act as key susceptibility factors of maize to *Colletotrichum graminicola* by suppressing salicylic acid‐mediated defence during the biotrophic phase of growth.

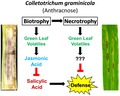

## 1 INTRODUCTION

*Colletotrichum* is one of the most widespread and prolific genera of plant pathogenic fungi in the world (Dean *et al*., 2012). *Co* *lletotrichum graminicola* is an economically relevant representative of this genus, and is one of the greatest sources of yield loss among maize pathogens (Mueller *et al*., 2016). Though *C. graminicola* infects several tissues of maize, its most devastating forms of disease arise from infection of stalks and leaves, which result in anthracnose stalk rot (ASR) and anthracnose leaf blight (ALB). Infection begins with germination of conidia 12 hr after contact with the plant surface and is followed by formation of melanized appressoria within 24 hr (Vargas *et al.*, 2012). A penetration peg is then formed after 24–36 hr, which leads to a phase of biotrophic colonization by primary hyphae (Mims and Vaillancourt, 2002; Vargas *et al.*, 2012). After approximately 48–72 hr the fungus switches to a phase of necrotrophic growth. This is characterized by the formation of thinner secondary hyphae that kill cells prior to infection, which results in the formation of necrotic lesions on infected tissues (O’Connell *et al*., 1985; Bergstrom and Nicholson, 1999; Wharton *et al.*, 2001; Mims and Vaillancourt, 2002; Vargas *et al.*, 2012). Though disease progression of *C. graminicola* is well documented, less is known regarding defences employed by maize against it. Existing literature implicates salicylic acid (SA), jasmonic acid (JA), and various other metabolites as potential regulators of defence against *C. graminicola*; however, genetic evidence has not yet been provided to validate these hypotheses (Vargas *et al.*, 2012; Balmer *et al.*, 2013; Miranda *et al*., 2017).

JA and green leaf volatiles (GLVs) are well‐known oxylipins produced in the lipoxygenase (LOX) pathway (Feussner and Wasternack, 2002; Andreou and Fuessner, 2009; Borrego and Kolomiets, 2016). JA biosynthesis in the allene oxide synthase (AOS) pathway begins in the chloroplast with the oxygenation of C18:3 by a 13‐LOX to form 13*S*‐hydroperoxy octadecatrienoic acid (13*S*‐HPOTE) (Blée, 2002; Howe and Shilmiller, 2002). 13*S*‐HPOTE is subsequently acted upon by a 13‐AOS and then an allene oxide cyclase (AOC) to form (+)‐*cis*‐12‐oxo‐phytodienoic acid (12‐OPDA), which possesses signalling activity distinct from JA (Vick and Zimmerman, 1983; Kramell *et al.*, 2000; Stintzi *et al.*, 2001; Taki *et al.*, 2005; Ribot *et al.*, 2008; Dave *et al.*, 2011; Dave and Graham, 2012). 12‐OPDA is then transported to the peroxisome, where it is reduced to 8‐[3‐oxo‐2‐*cis*‐[(*Z*)‐2‐pentenyl]cyclopentyl]octanoic acid (OPC‐8:0) by an oxo‐phytodienoate reductase (OPR). Maize possesses two functionally redundant JA‐producing OPRs, ZmOPR7 and ZmOPR8 (Yan *et al.*, 2012). OPC‐8:0 undergoes three rounds of β‐oxidation to form (+)‐7‐*iso*‐JA, which itself is biologically inactive (Staswick and Tiryaki, 2004). Once synthesized, JA may be converted into a variety of derivatives, including methyl jasmonate (MeJA) and the main biologically active conjugate (+)‐7‐*iso*‐JA‐Ile (JA‐Ile) (Vick and Zimmerman, 1983; Staswick and Tiryaki, 2004; Fonseca *et al.*, 2009; Yan *et al.*, 2016; Caarls *et al.*, 2017; Wasternack and Strnad, 2018). JA signalling regulates many diverse physiological processes and is a key regulator of the defence response to wounding, herbivory, and pathogen infection via induction of defensive metabolites and proteolytic enzymes (Farmer and Ryan, 1990; Rodriguez‐Saona *et al.*, 2001; Howe and Jander, 2008) or emission of volatile organic compounds (VOCs), such as GLVs (Kessler and Baldwin, 2001), which recruit insect parasitoids (Turlings *et al.*, 1995; Allman and Baldwin, 2010).

GLVs are an important class of VOC and are produced in the hydroperoxide lyase (HPL) pathway, functioning in inter‐ and intraplant signalling, plant–insect communication, and defence against a variety of environmental stresses. GLVs include C_6_ aldehydes, alcohols, and their corresponding esters, and are almost ubiquitously emitted in response to abiotic and biotic stresses. As with JA, GLV biosynthesis begins in chloroplasts with oxygenation of C18:3 by a 13‐LOX to produce 13*S‐*HPOTE (Blée, 2002; Howe and Schilmiller, 2002; Borrego and Kolomiets, 2016). Maize possesses a single LOX isoform, LOX10, that supplies substrate to the HPL pathway for GLV biosynthesis (Christensen *et al.*, 2013). A 13‐HPL acts upon LOX10‐derived 13*S*‐HPOTE to form a short‐lived hemiacetal, which quickly splits into C_6_ GLVs and traumatin, a C_12_ compound (Mukhtarova *et al.*, 2018). Exposure to GLVs induces broad‐spectrum defence to various stresses through up‐regulation of a variety of defence‐related genes, including genes involved in the biosynthesis and signalling of JA (Bate and Rothstein, 1998; Engelberth *et al.*, 2004; 2007; 2013; Farag *et al.*, 2005; Frost *et al.*, 2008; Hirao *et al.*, 2012; Christensen *et al.*, 2013; Yamauchi *et al.*, 2015). In addition to altering plant responses, GLVs can also directly inhibit growth of fungal and bacterial pathogens (Major *et al.*, 1960; Zeringue and McCormick, 1989; Hamilton‐Kemp *et al.*, 1992; Nakamura and Hatanaka, 2002; Prost *et al.*, 2005; Kishimoto *et al.*, 2006; Shiojiri *et al.*, 2006).

Thus far, the effects of GLVs on plant–pathogen interactions have almost exclusively been studied in the *Arabidopsis–Botrytis cinerea* pathosystem, where GLVs induce resistance (Archbold *et al.*, 1997, Kishimoto *et al.*, 2006; 2008; Shijori *et al*., 2006). In this pathosystem, defence conferred by GLVs is probably mediated through increased JA‐dependent signalling, which typically induces resistance to necrotrophic pathogens (Thomma *et al.*, 1998). JA shares strong antagonism with another important defence phytohormone, SA, which typically induces resistance to (hemi)biotrophic pathogens (Glazebrook, 2005). Using the maize GLV‐deficient mutant *lox10* and the JA‐deficient double mutant *opr7-5 opr8-2*, we show that GLVs facilitate susceptibility to *C. graminicola* through JA‐dependent suppression of SA shortly after inoculation.

## 2 RESULTS

### 2.1 GLV and JA deficiency results in increased resistance to ASR

**Figure 1:**
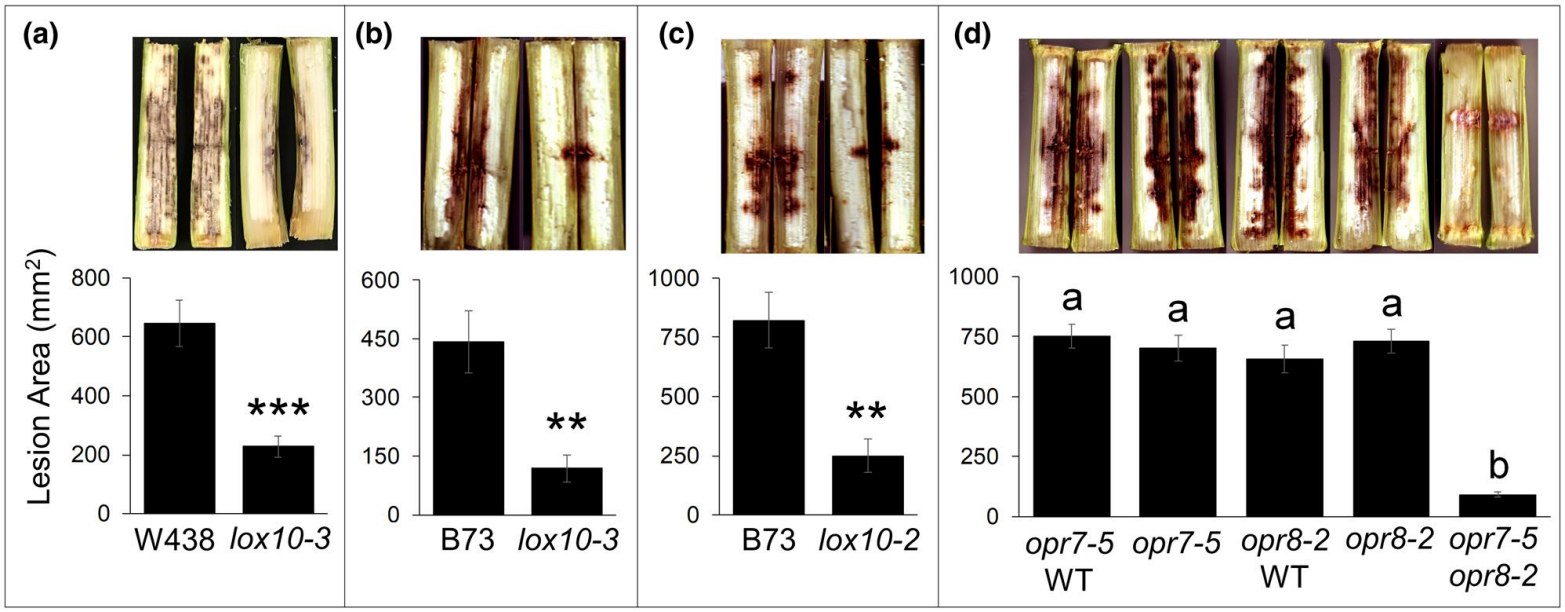
*lox10* and *opr7‐5 opr8‐2* mutant stalks are more resistant to *Colletotrichum graminicola*. (a) Representative stalks of wild‐type (WT) and *lox10‐3* mutants in the W438 genetic background 11 days post‐inoculation (dpi). (b) *lox10‐2* and (c) *lox10‐3* mutants and their near‐isogenic WTs in the B73 genetic background at 10 dpi. (d) Stalks of *opr7‐5*, *opr8‐2*, and *opr7‐5 opr8‐2* mutants and their respective WTs in the B73 background at 10 dpi. Stalks were split and imaged and lesions were quantified from the digital images. Mean ± *SE*, mm^2^. For (a), (b), and (c) Student's *t* test was used to determine statistical significance (**p < .005, ***p < .0005). Tukey's HSD test was used to determine the statistical significance for (d), where different letters denote statistical difference (*p* < .05)

To investigate the roles of GLVs and JA in ASR, we inoculated stalks of *lox10* mutants in two different genetic backgrounds, W438 and B73, with *C. graminicola*. Mutants displayed significantly smaller lesions relative to their respective near‐isogenic line wild types (NIL‐WTs), indicating increased resistance to ASR (Figure 1a–c). Furthermore, mutants in both genetic backgrounds were equally resistant, indicating LOX10 function in mediating susceptibility is well‐conserved across diverse genetic backgrounds (Figure 1a–c). Because GLVs are one of the major LOX10‐derived products and are well‐known inducers of JA synthesis (Engelberth *et al.*, 2004), we hypothesized that the increased resistance in *lox10* mutants is due to reduced JA. To test whether JA is a susceptibility factor for ASR, we inoculated stalks of single *opr7‐5* and *opr8‐2* mutants, *opr7‐5 opr8‐2* double mutants, and their respective NIL‐WTs. Of these genotypes, only the *opr7‐5 opr8‐2* double mutant is devoid of JA, while *opr7‐5* and *opr8‐2* single mutants maintain WT levels of JA due to functional redundancy of the *OPR7* and *OPR8* genes (Yan *et al.*, 2012). Accordingly, only *opr7‐5 opr8‐2* double mutants displayed increased resistance against ASR, whereas *opr7‐5* and *opr8‐2* single mutants and their respective NIL‐WTs all exhibited equally large lesions (Figure 1d). Taken together, these results imply that *lox10* and *opr7‐5 opr8‐2* mutant resistance is due to reduced GLV and JA biosynthesis.

### 2.2 Increased resistance to ASR correlates with reduced JA and increased SA

**Figure 2:**
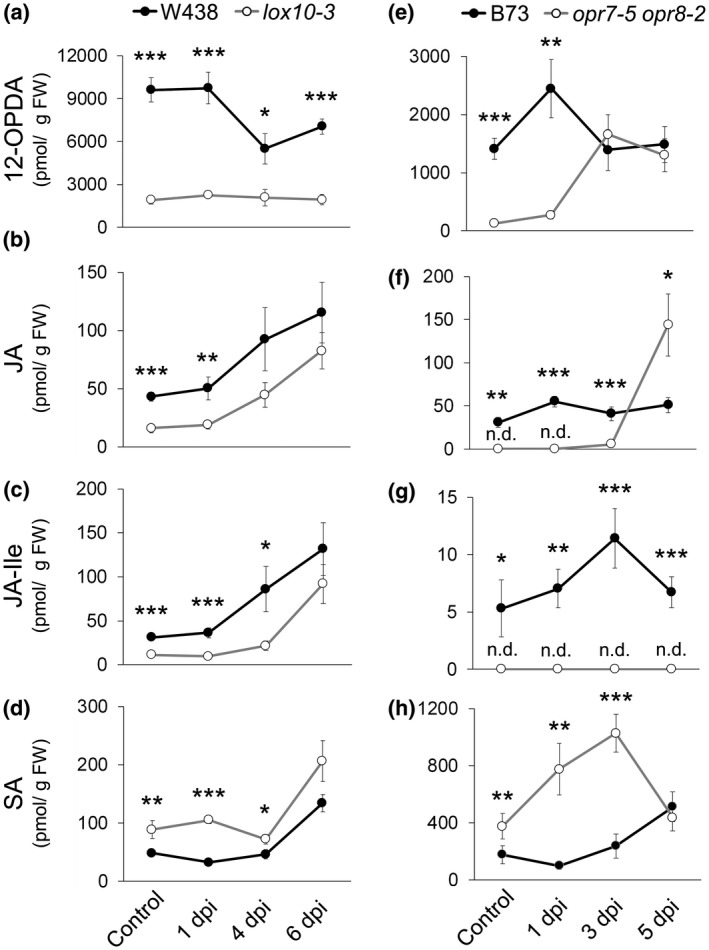
Hormone analysis shows 12‐OPDA, jasmonic acid (JA), and JA‐Ile are largely low or absent and salicylic acid (SA) is high in *lox10‐3* and *opr7‐5 opr8‐2* mutant stalks after inoculation. The left column shows 12‐OPDA (a), JA (b), JA‐Ile (c), and SA (d) levels in wild‐type (WT) and *lox10‐3* mutant stalks in the W438 background 1, 4, and 6 days post‐inoculation (dpi). The right column shows 12‐OPDA (e), JA (f), JA‐Ile (g), and SA (h) levels in WT and *opr7‐5 opr8‐2* mutants stalks in the B73 background 1, 3, and 5 dpi. n.d., not detected. Control = mock‐treated 1 dpi. Mean ± *SE*, pmol per g of fresh weight. Student's *t* test was used to determine the statistical difference between genotypes of each timepoint/treatment (*p < .05, **p < .005, ***p < .0005)

To investigate the biochemical mechanisms behind increased resistance of both GLV‐ and JA‐deficient mutants, we used liquid chromatography tandem mass spectrometry (LC‐MS/MS) to quantify accumulation of diverse hormones and metabolites, including SA, and the jasmonates 12‐OPDA, JA, and JA‐Ile during ASR progression in *lox10‐3* and *opr7‐5 opr8‐2* mutant stalks. *lox10‐3* mutants were significantly impaired in their ability to accumulate 12‐OPDA throughout the course of infection, as well as in uninoculated controls (Figure 2a). Similarly, *opr7‐5 opr8‐2* mutants accumulated low levels of 12‐OPDA at 1 day post‐inoculation (dpi) (Figure 2e). Unlike in *lox10‐3* mutants, however, levels of 12‐OPDA in *opr7‐5 opr8‐2* mutants recovered to WT levels by 3 dpi, presumably due to increased synthesis of 12‐OPDA by LOX10. This suggests that LOX10 is a major producer of 12‐OPDA in stalks.

JA and JA‐Ile accumulation were also impaired in *lox10‐3* mutants but, as in WT stalks, steadily increased throughout the duration of the experiment (Figure 2b,c). As expected, *opr7‐5 opr8‐2* mutants are largely devoid of JA and JA‐Ile, with the notable exception of 5 dpi, where JA, but not JA‐Ile, exceeded levels seen in WT stalks (Figure 2f,g). This induction of JA was unexpected, as *opr7‐5 opr8‐2* mutants were thought to be JA‐deficient (Yan *et al.*, 2012). However, many fungi are able to directly synthesize JA themselves (Tsukada *et al.*, 2010; Oliw and Hamberg, 2019), so we hypothesized that JA detected in *opr7‐5 opr8‐2* mutants was synthesized and secreted by *C. graminicola* in order to reduce host defences. To test this hypothesis, we measured jasmonates in *C. graminicola* mycelial mass filtered from liquid culture, as well as the liquid medium itself. Much to our surprise, no jasmonates of any kind were detected in either *C. graminicola* biomass or the liquid medium in which it was grown (data not shown).

Metabolite analysis revealed that SA was elevated in *lox10‐3* and *opr7‐5 opr8‐2* mutant stalks relative to their respective WTs (Figure 2d,h). Both mutants displayed elevated levels of SA at 1 dpi relative to their respective WTs. *opr7‐5 opr8‐2* mutants were particularly high, which is probably a result of them possessing higher basal SA, a result not observed in *lox10‐3* mutants (Figures 2d,h and S2). Importantly, the most significant differences of jasmonates and SA between both mutants and their respective WTs occurred at the earliest timepoints of infection, which coincide with a phase of biotrophic growth by *C. graminicola* (Figure 2). Overall, *lox10‐3* and *opr7‐5 opr8‐2* mutant resistance seems to correlate with increased SA and decreased JA in stalks at early stages of infection.

### 2.3 GLV and JA deficiency results in increased resistance to ALB

**Figure 3:**
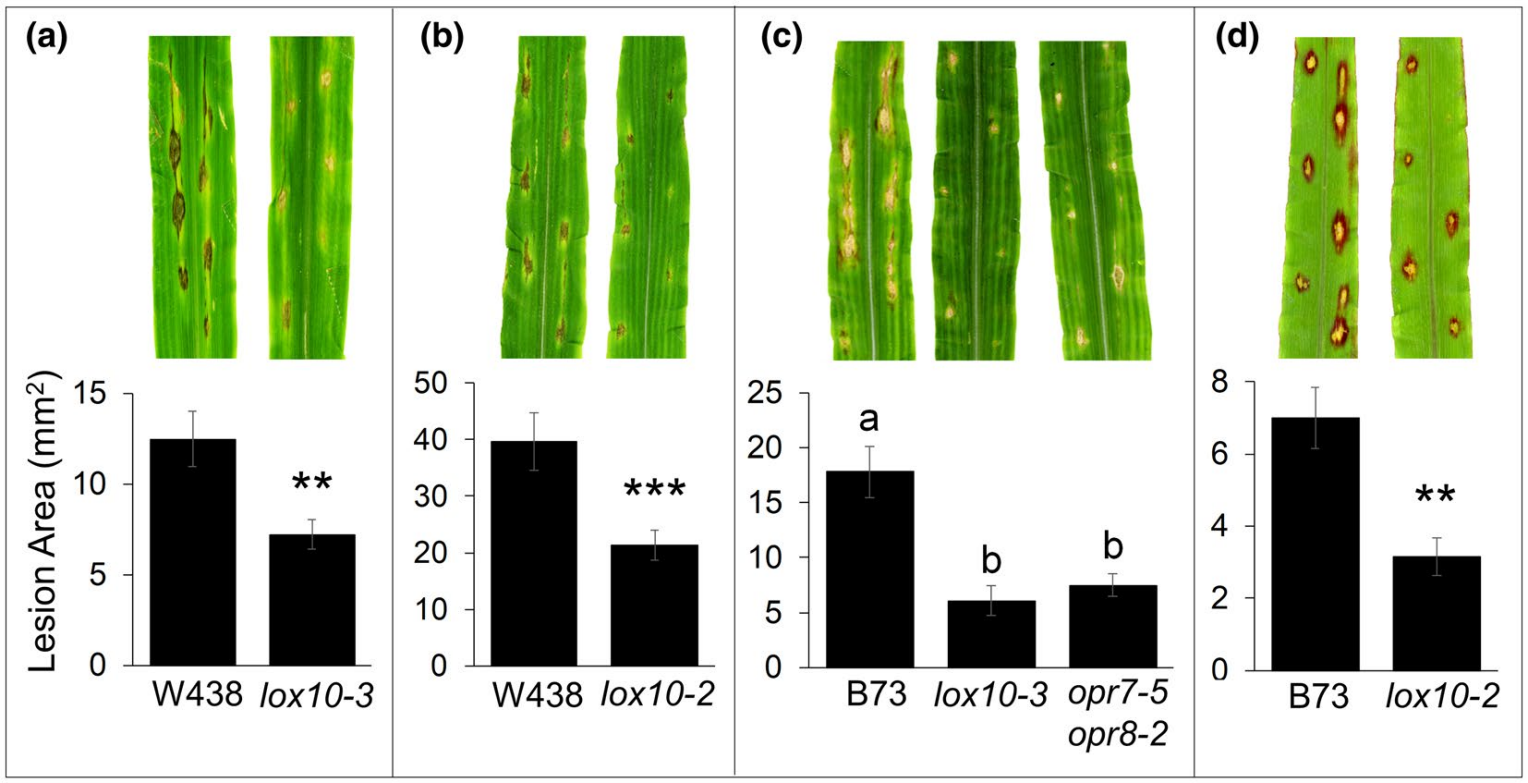
*lox10* and *opr7‐5 opr8‐2* mutant leaves are resistant to *Colletotrichum graminicola.* Mean areas of lesion after inoculation of *lox10‐2* (b) and (d), *lox10‐3* (a) and (b), and *opr7‐5 opr8‐2* (b) mutants. (a) and (b) *lox10‐2* and *lox10‐3* mutants in the W438 background. (c) and (d) *lox10‐2*, *lox10‐3*, and *opr7‐5 opr8‐2* mutants in the B73 background. Leaves were harvested at 5 days post‐inoculation (dpi) (a) and (c) or 6 dpi (b) and (d), and scanned to produce digital images from which lesion areas were measured. Mean ± *SE*, lesion area (mm^2^). For (a), (b), and (d), Student's *t* test was performed to determine the statistical significance of lesion areas (**p < .005, ***p < .0001). Tukey's HSD test was performed for (c), where different letters denote statistical significance (*p* < .05)

To test whether GLVs and JA mediate susceptibility to *C. graminicola* in leaves, we drop‐inoculated *C. graminicola* spore suspension onto leaves of *lox10‐2* and *lox10‐3* mutants in the W438 and B73 backgrounds, as well as *opr7‐5 opr8‐2* mutants in the B73 background. Similar to ASR, ALB assays consistently showed that *lox10‐2* and *lox10‐3* mutant leaves in both genetic backgrounds displayed significantly smaller lesions compared with their WT counterparts (Figure 3). Additionally, *opr7‐5 opr8‐2* mutant leaves also had significantly smaller lesions compared with WT (Figure 3c), and in direct side‐by‐side comparisons were similar to lesions on *lox10‐3* mutant leaves. Overall, this confirms that, as with ASR, GLVs and/or other LOX10‐derived products and JA promote virulence of *C. graminicola*.

### 2.4 Increased resistance to ALB correlates with reduced JA and increased SA

**Figure 4:**
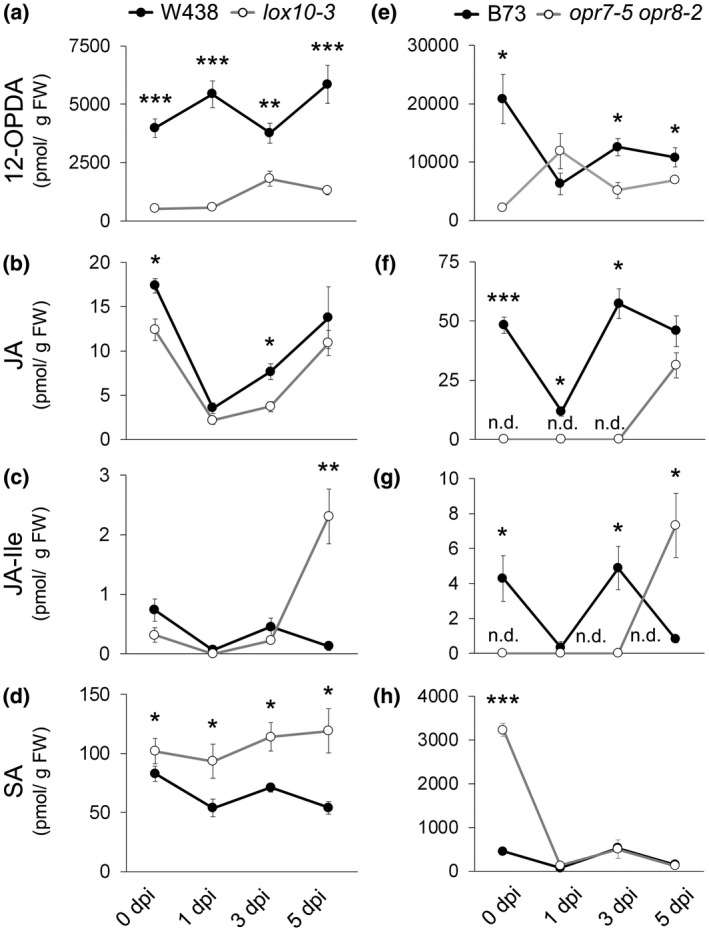
Hormone analysis shows 12‐OPDA, jasmonic acid (JA), and JA‐Ile are largely low or absent, and that salicylic acid (SA) is high in *lox10‐3* and *opr7‐5 opr8‐2* mutant leaves before or throughout infection. The left column shows 12‐OPDA (a), JA (b), JA‐Ile (c), and SA (d) levels in wild‐type (WT) and *lox10‐3* mutants leaves in the W438 background 0, 1, 3, and 5 days post‐inoculation (dpi). The right column shows 12‐OPDA (e), JA (f), JA‐Ile (g), and SA (h) levels in WT and *opr7‐5 opr8‐2* mutant leaves in the B73 background at 0, 1, 3, and 5 dpi. 0 dpi plants were untreated. Mean ± *SE*, pmol per g of fresh weight. n.d., not detected. Student's *t* test was used to determine the statistical difference between genotypes of each timepoint/treatment (*p < .05, **p < .005, ***p < .0005)

To determine if the same mechanisms behind ASR resistance of *lox10‐3* and *opr7‐5 opr8‐2* mutant stalks also underlie ALB resistance in leaves, we quantified 12‐OPDA, JA, JA‐Ile, and SA throughout progression of ALB in leaves of *lox10‐3* and *opr7‐5 opr8‐2* mutants. As in stalks, 12‐OPDA and JA levels were low in *lox10‐3* mutants throughout the duration of disease progression (Figure 4a). JA‐Ile largely mimicked this same pattern, but by 5 dpi, *lox10‐3* mutants accumulated more JA‐Ile than WT (Figure 4c). *opr7‐5 opr8‐2* mutant leaves, with the exception of 1 dpi, displayed lower amounts of 12‐OPDA compared with WT, similar to results obtained for stalks (Figure 4e). Unsurprisingly, *opr7‐5 opr8‐2* mutant leaves were devoid of JA or JA‐Ile throughout 3 dpi (Figure 4f,g), but at 5 dpi both accumulated to levels greater than seen in WT (Figure 4f,g). This late and robust accumulation of JA in *opr7‐5 opr8‐2* mutant leaves at 5 dpi mirrors results in stalks, but unlike in stalks of *opr7‐5 opr8‐2* mutants, also includes a proportionate increase of JA‐Ile. Interestingly, this late increase in JA and/or JA‐Ile was seen in leaves of both *lox10‐3* and *opr7‐5 opr8‐2* mutants, which characteristically produce low amounts of JA. These results again suggest potential differing roles of JA during biotrophic and necrotrophic phases of growth.

As in stalks, SA is elevated in *lox10‐3* mutant leaves throughout the course of infection (Figure 4d). In *opr7‐5 opr8‐2* mutant leaves, SA levels were equal to WT throughout infection (Figure 4h). This was surprising given the high amounts of SA seen in *opr7‐5 opr8‐2* mutant stalk tissues. However, it is important to note that the levels of SA prior to infection (0 dpi) were strikingly higher relative to WT. Furthermore, the levels of SA in leaves of *opr7‐5 opr8‐2* mutant were much higher compared to the amounts detected in their stalks. This suggests that high basal amounts of SA present in *opr7‐5 opr8‐2* mutant leaves may prevent initial colonization by *C. graminicola*, resulting in limited infection and thus limited defence induction. Conversely, *lox10‐3* mutants possessed high amounts of SA after infection, but, as in stalks, had normal basal levels of SA. This suggests that although the mechanisms behind SA‐related defence in *lox10‐3* and *opr7‐5 opr8‐2* mutant leaves and stalks are different, both mutants are resistant due to enhanced amounts of SA. Taken in concert with the relatively low amount of jasmonates present in both mutants, especially at the earliest points of infection, this suggests that early LOX10‐, OPR7‐, and OPR8‐dependent accumulation of JA suppresses SA‐mediated defence against *C. graminicola*. However, in addition to producing potent JA‐inducing GLVs, LOX10 may also provide substrate for synthesis of a variety of other metabolites, including JA (Borrego and Kolomiets, 2016). As such, it was unclear whether LOX10 increases JA through GLV‐dependent induction, direct synthesis of JA, both, or other LOX10‐derived metabolites.

### 2.5 LOX10 localizes to chloroplasts, the site of JA and GLV biosynthesis

**Figure 5:**
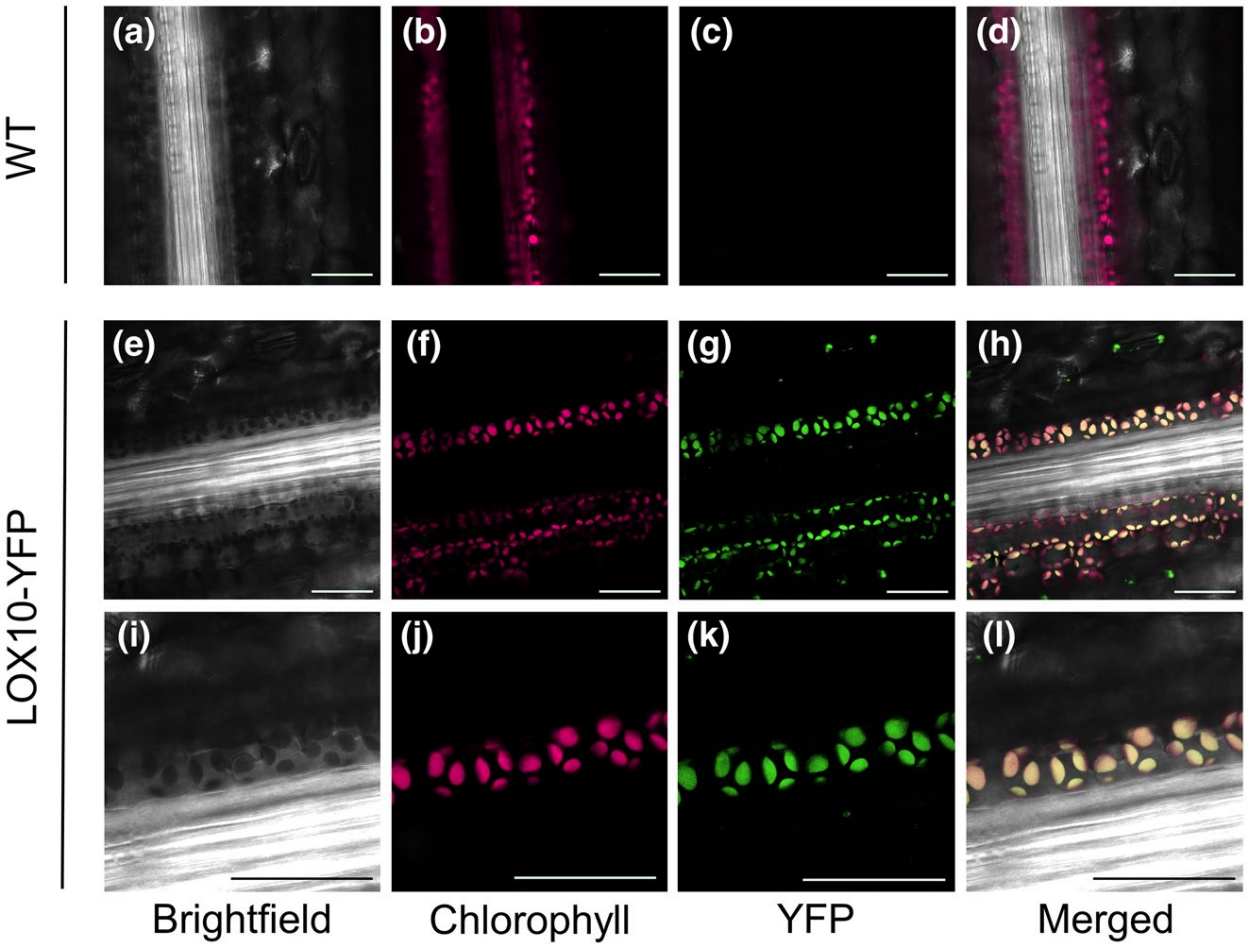
LOX10‐yellow fluorescent protein (YFP) tagged maize lines reveal that LOX10 localizes to chloroplasts of bundle sheath cells. (a)–(d) Images of untransformed leaves of B73 inbred line, (e)–(l) images of transgenic lines in the B73 background expressing YFP‐tagged LOX10 (LOX10‐YFP) under its native promoter, where (e)–(h) show images comparable to (a)–(d) and (i)–(l) show zoomed‐in views of (e)–(h) detailing LOX10 localization to bundle sheath chloroplast (i)–(l). Columns in order of left to right show brightfield views, chlorophyll autofluorescence, YFP fluorescence, and a merged view. Scale bars = 50 µm

We first sought to establish whether LOX10 localizes to chloroplasts, the known site of both GLV and JA biosynthesis, as with other GLV‐producing LOXs (Bell *et al.*, 1995; Chehab *et al.*, 2006; Shen *et al.*, 2014; Mochizuki *et al.*, 2016). Using maize containing yellow fluorescent protein (YFP)‐tagged LOX10 (LOX10‐YFP), Christensen *et al. *(2013) previously reported that LOX10 localized to unknown microbodies but its localization to plastids was uncertain. To further understand LOX10 localization, we performed additional confocal microscopy on LOX10‐YFP lines and found LOX10 also localized to chloroplasts (Figure 5). LOX10 seemed to accumulate most prominently in chloroplasts of bundle sheath cells and guard cells, as well as in chloroplasts of mesophyll cells, albeit to a lesser degree. These results suggest that 13*S*‐HPOTE produced by LOX10 may potentially feed into the AOS pathway for JA synthesis as well as into the HPL pathway for GLV synthesis.

### 2.6 GLVs mediate susceptibility to *C. graminicola* through induction of JA

**Figure 6:**
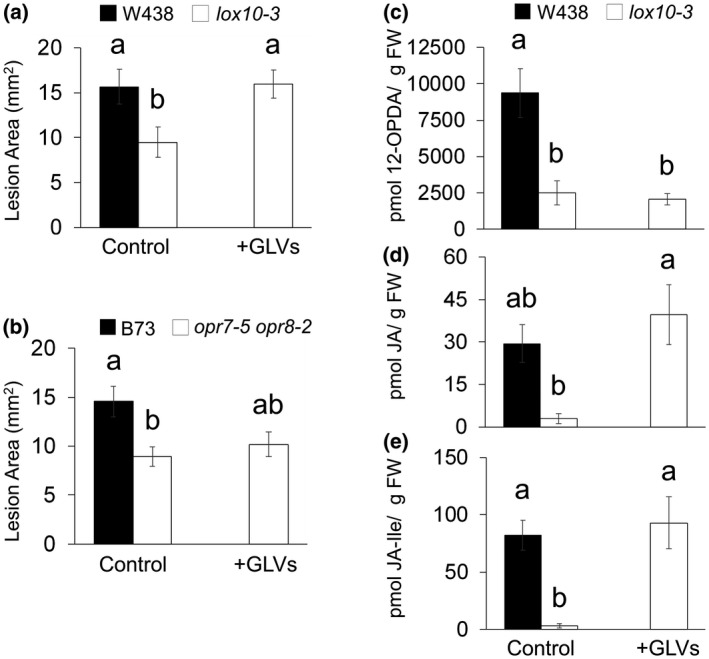
Green leaf volatiles (GLVs) exposure rescues *lox10‐3* mutant*,* but not *opr7‐5 opr8‐2* mutant, susceptibility by increasing jasmonic acid (JA) and JA‐Ile. *lox10‐3* and *opr7‐5 opr8‐2* mutants in the W438 (a) or B73 (b) backgrounds were exposed to either an exogenous GLV mixture or triacetin (control) for 1 hr, followed by inoculation with *Colletotrichum graminicola*. Infected leaves were harvested and scanned at 4 days post‐inoculation to produce digital images from which lesions were measured. Mean ± *SE*, mm^2^. 12‐OPDA (c), JA (d), and JA‐Ile (e) were measured in *lox10‐3* mutants after GLV exposure. Mean ± *SE*, pmol per g of fresh weight. Tukey's HSD test was used to determine the statistical significance, where different letters denote statistical significance (*p* < .05)

To uncover the mechanism behind LOX10‐mediated JA induction, we exposed *lox10‐3* and *opr7‐5 opr8‐2* mutants to exogenous GLV treatment for 1 hr prior to inoculation of their leaves. Treatment consisted of a mixture containing 10 nmol of each major GLV molecular species emitted by maize, (*Z*)‐3‐hexenal, (*Z*)‐3‐hexenol, and (*Z*)‐3‐hexenyl acetate. Exposure of *lox10‐3* mutants to GLVs resulted in lesion sizes equal to those observed on WT controls, and were significantly larger than those on *lox10‐3* mutant controls (Figure 6a). In contrast, *opr7‐5 opr8‐2* mutants exposed to GLVs did not display increased susceptibility compared to *opr7‐5 opr8‐2* mutant controls (Figure 6b). These results show that small amounts of GLVs can completely rescue *lox10‐3* mutant resistance, but are unable to rescue JA‐deficient *opr7‐5 opr8‐2* mutants (Figure 6a,b).  This confirms that GLVs are a major susceptibility factor and suggests that GLVs act through JA to induce susceptibility.

To confirm that rescue of *lox10‐3* mutants by GLV exposure is mediated by increased JA, we exposed *lox10‐3* mutants to GLVs and quantified 12‐OPDA, JA, and JA‐Ile. Accumulation of these jasmonates was significantly diminished in *lox10‐3* mutant controls compared to WT controls; however, JA and JA‐Ile levels in *lox10‐3* mutants were completely restored after GLV exposure (Figure 6c,d,e). Interestingly, 12‐OPDA was not increased by GLV exposure (Figure 6c). This could be due to GLV induction of genes in the AOS pathway downstream of 12‐OPDA synthesis, such as OPR7 and OPR8 (Christensen *et al.*, 2013), and/or LOX10 is the major producer of 12‐OPDA. These results confirm that LOX10‐derived GLVs induce JA in a LOX10‐independent manner and are able to fully rescue both JA levels and susceptibility in *lox10-3* mutants. Collectively, these data support the hypothesis that GLVs increase susceptibility to *C. graminicola* through induction of JA, and thus suppression of SA.

### 2.7 SA induces defence and JA promotes susceptibility to *C. graminicola*

**Figure 7:**
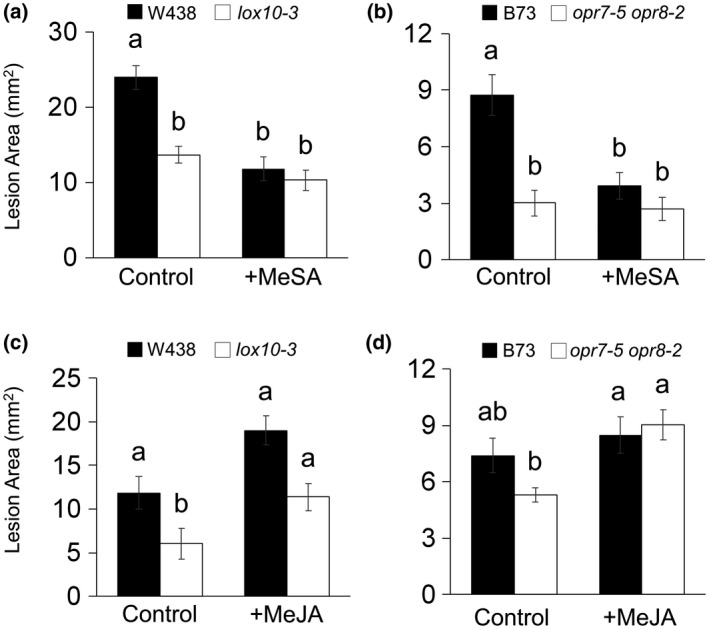
Treatment with methyl jasmonate (MeJA) rescues susceptibility in *lox10‐3* and *opr7‐5 opr8‐2* mutants while treatment with methyl salicylate (MeSA) has no effect. Wild type (WT) and *lox10‐3* mutant in the W438 genetic background (a) and (b), and WT and *opr7‐5 opr8‐2* mutants in the B73 genetic background were exposed to MeSA (a) and (b), MeJA (c) and (d), or ethanol (control) before inoculation with *Colletotrichum graminicola*. Treatments consisted of exposing plants to 10 µmol MeSA or MeJA dissolved in ethanol, or ethanol (control) for 2 days post‐inoculation (dpi) (a) and (b) or 6 dpi (c), (d), and (h). Leaves were harvested and scanned at 4 dpi to produce digital images from which lesions were measured. Mean ± *SE*, mm^2^. Tukey's HSD test was used to determine statistical significance, where different letters denote statistical significance (*p* < .05)

SA is commonly required for resistance to biotrophs or hemibiotrophs and is implicated in systemic acquired resistance (SAR) to *C. graminicola* in maize (Balmer *et al.*, 2013), but the roles of JA and SA in local infections remain untested. To confirm these respective roles, we exposed WT, *lox10‐3*, and *opr7‐5 opr8‐2* mutants to methyl salicylate (MeSA) or methyl jasmonate (MeJA) prior to infecting their leaves with *C. graminicola*. WT plants in both the W438 and B73 backgrounds exposed to MeSA exhibited significantly smaller lesions compared with their respective controls, and were comparable to *lox10‐3* and *opr7‐5 opr8‐2* mutant controls (Figure 7a,b). In contrast, WT plants in the W438 background exposed to MeJA exhibited larger lesions than WT controls (Figure 7c). This increased susceptibility due to exogenous MeJA was not seen in the B73 background (Figure 7d). MeSA was unable to further decrease lesion sizes in *lox10‐3* or *opr7‐5 opr8‐2* mutants compared to their respective controls, presumably due to their innate saturation of SA‐dependent defences (Figure 7a,b). These results conclusively showed that SA induces resistance and JA promotes susceptibility to *C. graminicola* during its biotrophic phase of growth, and confirmed that high SA and low JA early in the infection process are responsible for *lox10‐3* and *opr7‐5 opr8‐2* mutant resistance.

### 2.8 GLVs, but not JA, induce susceptibility after the switch to necrotrophy by *C. graminicola*

**Figure 8:**
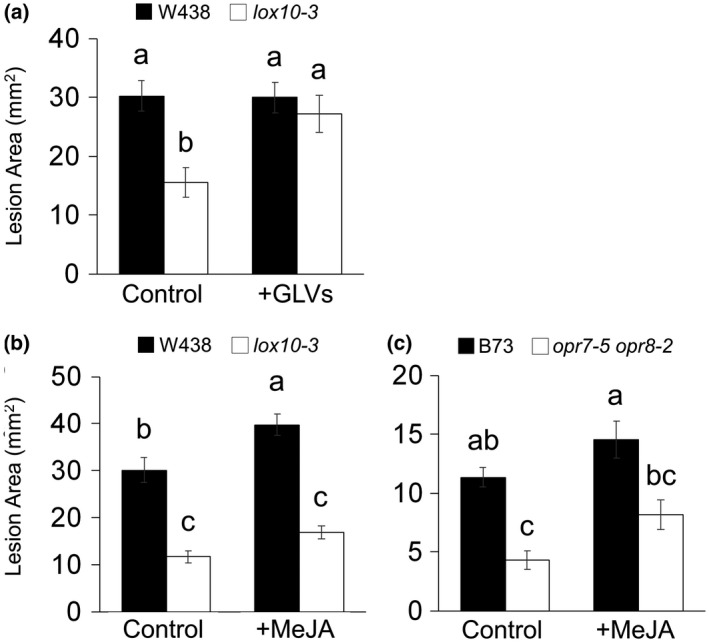
Green leaf volatiles (GLVs) treatment during necrotrophy rescued susceptibility in *lox10‐3* mutants, but methyl jasmonate (MeJA) treatment did not. *lox10‐3* and *opr7‐5 opr8‐2* mutants were inoculated with *Colletotrichum graminicola* and infection was left to proceed for 3 days before they were exposed to GLVs (a), MeJA (b) and (c), and their respective control treatments (triacetin and ethanol, respectively). Leaves were harvested and scanned 6 days post‐inoculation to produce digital images from which lesions were measured. Mean ± *SE*, mm^2^. Tukey's HSD test was used to determine statistical significance, where different letters denote statistical significance

As GLVs and JA are known to aid in defence against necrotrophic pathogens, we sought to better clarify the roles of these metabolites after the switch to necrotrophy. To determine this, leaves of *lox10‐3* and *opr7‐5 opr8‐2* mutants were inoculated and infection was allowed to proceed for 3 days. At 3 dpi and the formation of visible lesions, we exposed the infected plants to either GLVs or MeJA and leaves were harvested at 6 dpi. As with the treatment before inoculation, GLV treatment during necrotrophy was able to fully rescue WT levels of susceptibility in *lox10‐3* mutants (Figure 8a). However, MeJA treatment only slightly, but not significantly, enhanced susceptibility of *lox10‐3* and *opr7‐5 opr8‐2* mutants (Figure 8b,c). This indicates that JA only significantly contributes to disease progression during the earliest points of infection, when *C. graminicola* grows biotrophically, but not during the necrotrophic phase of growth. Furthermore, this indicates that while GLVs rely on JA induction for susceptibility during biotrophic growth, these molecules contribute to susceptibility through unknown, JA‐independent mechanisms during necrotrophic growth.

## 3 DISCUSSION

Inoculation of stalks and leaves of GLV‐deficient *lox10* mutants and JA‐deficient *opr7‐5 opr8‐2* mutants clearly revealed that LOX10 and JA are susceptibility factors during *C. graminicola* infection (Figures 1 and 4). Importantly, resistance was observed in *lox10‐2* and *lox10‐3* mutants in both the B73 and W438 genetic backgrounds, indicating that LOX10‐mediated susceptibility is conserved across diverse genetic backgrounds in leaves and stalks. As LOX10, OPR7, and OPR8 are involved in the synthesis and/or induction of JA, it was not surprising that *lox10‐3* and *opr7‐5 opr8‐2* mutant stalks and leaves displayed impaired accumulation of several jasmonates during the early stages of infection. However, we were surprised to find that *lox10‐3* mutant leaves and *opr7‐5 opr8‐2* mutant leaves and stalks experienced increased amounts of JA at later stages of infection, 5–6 dpi, after the switch to necrotrophy (Figures 3 and 5). Interestingly, *opr7‐5 opr8‐2* mutant leaves, but not stalks, also accumulated JA‐Ile at this time, which could be due to differences in JAR1 expression between the two tissue types. We hypothesized this JA was directly synthesized and secreted by *C. graminicola*, but jasmonates were not detected in analysis of either *C. graminicola* or its growth medium (data not shown). Despite this result, we cannot rule out that *C. graminicola* is directly producing this JA, as it may require physical or chemical cues and/or access to plant substrate from the plant surface to initiate JA biosynthesis. Alternatively, it is possible this JA was synthesized by the plant through a non‐OPR7/OPR8 dependent pathway, as has been reported for *opr3* mutants in *Arabidopsis* (Chini *et al.*, 2018). Despite its unknown origins, this late JA increase reveals a possible shift in the role of JA upon the switch from biotrophy to necrotrophy. Ultimately, however, *lox10‐3* and *opr7‐5 opr8‐2* mutant leaves and stalks possess low amounts of jasmonates during the early stages of infection, which are probably the most critical for establishing effective SA‐mediated defence.

In contrast to JA, *lox10‐3* and *opr7‐5 opr8‐2* mutants had higher amounts of SA in both stalks (Figure 3d,h) and leaves (Figure 4d,h). *lox10‐3* mutant stalks and leaves had similar levels of SA compared with WT prior to infection, but had significantly higher amounts as soon as 1 dpi (Figure 3d). Unlike *lox10‐3* mutants, stalks and leaves of *opr7‐5 opr8‐2* mutants had high basal amounts of SA compared to WT. As early as 1 dpi, stalks of *opr7‐5 opr8‐2* experienced even greater accumulation of SA relative to WT; however, the amount of SA in leaves of *opr7‐5 opr8‐2* mutants returned to WT levels after 1 dpi. This is probably because of the very high amounts of SA present in *opr7‐5 opr8‐2* mutant leaves, which were significantly higher than in stalks. This high basal amount may effectively prevent infection, thus resulting in diminished defence hormone induction. It should be noted that all leaves, but not stalks, of all genotypes experienced a decrease in most metabolites and hormones at 1 dpi. This is attributed to the inoculation method for leaves, which involved a 24 hr incubation in a humidity chamber with comparatively limited air flow and available light for photosynthesis. The relatively high amounts of SA present in both *lox10‐3* and *opr7‐5 opr8‐2* mutants before or soon after infection (1 dpi) directly coincide with appressoria formation and initiation of biotrophic growth by *C. graminicola* (Vargas *et al.*, 2012). Reactive oxygen species are rapidly produced by plants on infection via SA signalling and are involved in fungal defence and growth inhibition, including *C. graminicola* (Apostol *et al*., 1989; Mellersh *et al.*, 2002; Albarouki and Deising, 2013). It is possible that the quick induction of SA‐related defences inhibits catalase‐mediated degradation of H_2_O_2_, allowing build‐up of H_2_O_2_ around points of appressorial penetration, which stops penetration and/or growth of *C. graminicola*.

The mechanism behind low concentrations of JA was clear in *opr7‐5 opr8‐2* mutants, but less so in *lox10‐3* mutants as GLV‐producing LOXs are also usually the major JA‐producing LOXs. In maize, there are several LOXs that may directly contribute to JA synthesis, such as LOX8, a known producer of JA in response to wounding (Acosta *et al.*, 2009; Christensen *et al.*, 2013). As such, there was uncertainty as to whether LOX10 increases JA upon inoculation through direct synthesis, through GLV‐mediated signalling, both, or perhaps through other LOX10‐derived metabolites. Previous microscopy of LOX10‐YFP in maize did not show LOX10 localization to plastids, despite it containing a predicted plastid localization signal and LOX10 protein being previously isolated from chloroplasts (Majeran *et al.*, 2005; Christensen, *et al.*, 2013). As such, we first wanted to confirm if LOX10 localizes to chloroplasts, the initial site of both GLV and JA synthesis. These new results clearly detail LOX10 localization to chloroplasts of bundle sheath, mesophyll, and guard cells, indicating potential for JA biosynthesis (Figure 5 and S3). Importantly, LOX10‐YFP signal was distinctly more intense in bundle sheath chloroplasts compared to those of mesophyll cells, highlighting a potentially pivotal role in systemic vascular communication (Figure S3). These findings agree with analysis of LOX10 by ChloroP software, which predicts the presence of a 58 amino acid chloroplast transit peptide (Nemchenko *et al.*, 2006), and with prior proteome analysis revealing LOX10 is abundant in both mesophyll and bundle sheath plastids (Majeran *et al.*, 2005).

To uncover the mechanism behind LOX10‐mediated increase of JA on infection, we exposed *lox10‐3* and *opr7‐5 opr8‐2* mutants to GLVs prior to infection. GLV exposure of *lox10‐3* and *opr7‐5 opr8‐2* mutants resulted in a full rescue of susceptibility in *lox10‐3* mutants, but not *opr7‐5 opr8‐2* mutants (Figure 6a,b). Furthermore, GLV exposure fully restored JA and JA‐Ile in *lox10‐3* mutants (Figure 6c). This result agrees with previous analysis of JA in *lox10‐2* mutants after GLV exposure (Christensen *et al.*, 2013). This demonstrates that GLVs alone can induce susceptibility to *C. graminicola* and that GLV‐mediated susceptibility, at least during biotrophy, requires intact JA signalling. Furthermore, this shows that biologically relevant amounts of GLVs increase JA and susceptibility independent of direct LOX10 JA synthesis. Remarkably, 12‐OPDA levels were not rescued in *lox10‐3* mutants exposed to GLVs, suggesting that LOX10 is the major producer of 12‐OPDA. This also indicates that GLV‐mediated JA induction is achieved by activation of steps in the AOS pathway following AOC‐mediated synthesis of 12‐OPDA, implicating *OPR7* and *OPR8* as potential specific targets of GLVs for JA induction (Christensen *et al.*, 2013).

Exposure of *lox10‐3* and *opr7‐5 opr8‐2* mutants to MeJA fully restored WT levels of susceptibility in both mutants (Figure 7c,d), agreeing with the low levels of jasmonates detected in both mutants at early stages of infection. Conversely, MeSA exposure was unable to induce further resistance in either mutant, which is probably due to their already saturated SA‐dependent defence signalling before or on infection. Furthermore, MeSA was also able to induce resistance in WT on a par with either mutant and MeJA was able to induce further susceptibility of WT only in the W438 background, which could be due to underlying differences in the two backgrounds (Figure 7). These data provide strong genetic and chemical evidence that JA is a susceptibility factor, and that SA provides resistance to *C. graminicola*.

However, as both *lox10‐3* and *opr7‐5 opr8‐2* mutant leaves displayed low initial amounts of JA, followed by high amounts by 5 dpi, we sought to determine if the role of JA and GLVs changed after the shift from biotrophic growth to necrotrophic growth of *C. graminicola*. Exposure of *lox10‐3* and *opr7‐5 opr8‐2* mutants to MeJA after 3 days of infection, after the switch to necrotrophy, was not able to rescue susceptibility as with the treatment before inoculation (Figure 8b,c). This indicates that while JA initially acts as a susceptibility factor, its contribution to susceptibility becomes negligible after the fungus switches to a necrotrophic phase of growth. In contrast to JA, GLVs were still able to fully restore susceptibility in *lox10‐3* mutants after the switch to necrotrophy (Figure 8a). This shows that during necrotrophy, GLVs rely on other mechanisms than JA after the switch to necrotrophy. These mechanisms remain unknown and should be the subject of future studies. Furthermore, this shows that GLVs contribute to susceptibility in both phases of growth. This result contrasts with the previous study by Ameye *et al. *(2015), in which GLV treatment after the switch to necrotrophy by another hemibiotroph, *Fusarium graminearum*, in wheat induced resistance. The role GLVs play in plant–pathogen interactions may largely depend on the lifestyle of the pathogen; however, we should not discount the possibility that the effect of GLVs on plant–pathogen defence is pathosystem‐specific.

Little is known regarding maize defence mechanisms against *C. graminicola*. JA, SA, and other metabolites were suggested to be important for defence, but these hypotheses were not investigated with the use of knockout mutants or exogenous chemical treatment. Using JA‐ and GLV‐deficient mutants, we have definitively shown that JA, and GLVs by virtue of JA induction, cause maize susceptibility to *C. graminicola* in the biotrophic phase of growth. Much of this susceptibility can be attributed to JA‐mediated antagonism of SA‐related defences at this time, although there could be additional SA‐independent effects of JA or GLVs responsible as well. This is particularly true of GLV‐mediated susceptibility during necrotrophy. This suggests that LOX10, OPR7, OPR8, and other genes involved in synthesis or induction of GLVs and JA are potential targets for induction by *C. graminicola* shortly after colonization. It is important to note that herbivory and mechanical damage are strong inducers of GLVs and JA in maize (Christensen *et al.*, 2013) and that ASR frequency in field settings shows a direct correlation with herbivory by the European corn borer (ECB) (*Ostrinia nubilalis*) (Keller *et al.*, 1986; Bergstrom and Nicholson, 1999; Venard and Vaillancourt, 2007). This is in part because ECB can vector *C. graminicola* and allow it to bypass the tough rind of the stalk. However, this work reveals that a critical biochemical mechanism behind this correlation may also be the suppression of SA‐mediated defences by GLV and JA induction on herbivory (Bergstrom and Nicholson, 1999; Venard and Vaillancourt, 2007). This study also expands on the known functions of GLVs in plant–pathogen interactions, adding to a small number of publications that show GLVs can induce susceptibility to (hemi)biotrophs (Tong *et al.*, 2012; Scala *et al.*, 2013; Ameye *et al.*, 2015).

## 4 EXPERIMENTAL PROCEDURES

### 4.1 Plant and fungal material

As previously described, mutant alleles of *ZmLOX10*, *ZmOPR7*, and *ZmOPR8* were obtained by PCR screening of the Mutator‐transposon insertional genetics resource at DuPont‐Pioneer, Inc. (http://www.pioneer.com) for insertions in these genes (Yan *et al.*, 2012; Christensen *et al.*, 2013). The *lox10‐2*, *lox10‐3*, *opr7‐5*, and *opr8‐2* alleles are all confirmed exon‐insertional knockout mutants. Original mutants were backcrossed into the B73 (*lox10‐2*, *lox10‐3*, *opr‐5*, and *opr8‐2*) and W438 (*lox10‐2* and *lox10‐3*) backgrounds and genetically advanced to the backcross (BC) BC_5_ to BC_7_ stages. All mutants were genotyped by PCR analysis for conformation of homozygous mutant status. Transgenic C‐terminal YFP‐tagged LOX10 used in this study were generated as described by Mohanty *et al. *(2009) and advanced to the BC_3_ stage in the B73 genetic background. All *C. graminicola* plates used in infection assays were grown from culture stock (*C. graminicola* 1.001 strain) kept in a −80 °C freezer. Cultures were grown on potato dextrose agar (PDA) plates for at least 2 weeks before conidia were collected for use in plant inoculations. Spore extractions were performed as previously described by Gao *et al. *(2007) and were used within 2 hr of extraction.

### 4.2 Microscopy

LOX10‐YFP plants were grown to the V3 stage in TX‐360 Metro Mix soil (Sun Gro Horticulture) in a growth chamber under 16 hr of light (c.600 µmol⋅m^−2^⋅s^−1^) at 28 °C and 8 hr of dark at 24 °C with 50% humidity. Leaves were harvested and then imaged using a Digital Eclipse C1 confocal microscope (Nikon) (Tolley *et al.*, 2018).

### 4.3 Anthracnose leaf blight assays and volatile treatments

Plants used in ALB assays were grown as previously described to the V4 stage, and were inoculated as previously described by Gao *et al. *(2007) except six plants per genotype/treatment were inoculated at six different points. *opr7 opr8* mutants and their WT were grown in sterile soil, as they cannot survive in normal soil (Yan *et al.*, 2012). For lesion size determination, plants were left for 4–6 days after inoculation before the infected leaves were excised and scanned to produce digital images. Lesion sizes were determined from digital images using ImageJ software (Schneider *et al.*, 2012). For volatile treatments, plants were exposed to GLVs for 1 hr, MeSA for 2 hr, or MeJA for 6 hr before being inoculated 1 hr after volatile exposure ended. Six plants of each genotype/treatment were placed into a 6‐L glass container along with a cotton ball containing 100 µl of the chosen volatile(s). GLV treatment consisted of a mix containing 10 nmol (*Z*)‐3‐hexenal (50% in triacetin), 10 nmol (*Z*)‐3‐hexenol (>98%), and 10 nmol (*Z*)‐3‐hexenyl acetate (>98%,) dissolved in triacetin. MeSA (>98%) and MeJA (>98%) treatment consisted of 10 µmol of either chemical dissolved in ethanol. All chemical standards were purchased from Sigma‐Aldrich. For GLV and JA treatments during necrotrophy, plants were exposed to the volatile treatments at 3 dpi, and leaves were harvested for lesion size determination after 6 dpi.

### 4.4 Anthracnose stalk rot assays

*lox10‐2* and *lox10‐3* mutants and their NIL or inbred WTs used in ASR assays were grown to the VT stage outdoors under natural conditions throughout late spring and summer (College Station, TX, USA) in 14‐L pots filled with TX‐360 Metro Mix soil. Stalks were inoculated as previously described by Gao *et al. *(2007). For determination of lesion areas in *lox10* mutants, three internodes of 10 plants per genotype were infected and harvested at 10–11 dpi. Postharvest, infected internodes were split and photographed to produce digital images that were analysed by ImageJ software. *opr7‐5* and *orp8‐2* mutants were grown in 14‐L pots filled with sterile TX‐360 Metro Mix soil in greenhouses during the summer. Four internodes of four plants per genotype were inoculated, harvested, and analysed at 10 dpi as described above.

### 4.5 Hormone analysis

For analysis of hormones in response to GLV exposure, plants were grown in a growth chamber to the V4 stage and exposed to GLVs as previously described. Leaves of the plants were harvested, frozen in liquid N_2_, and stored in a −80 °C freezer. For hormone analysis ALB, shoots were inoculated using an atomizer to spray a mist of 5 ml of 10^6^ spores/ml onto each plant (0 hr timepoint plants were not treated) before plants were sealed in a humidity chamber as previously described by Gao *et al. *(2007). Five plants per genotype/timepoint were used. After 1, 3, or 5 dpi, their leaves were harvested, immediately frozen in liquid N_2_, and stored in a −80 °C freezer until further use. For hormone analysis of ASR, plant stalks were inoculated with either *C. graminicola* or sterile distilled water (control) as previously described. For *lox10‐3* mutants, three internodes of five plants per genotype/timepoint were inoculated and harvested 1, 4, and 6 dpi as described above. For *opr7‐5 opr8‐2* mutants, four internodes of four plants per genotype/timepoint were inoculated and harvested after 1, 3, or 5 dpi. A mortar and pestle were used to grind frozen plant material into a fine powder under liquid N_2_. Hormones were extracted from tissue and quantified by LC‐MS/MS. Ground tissue (100 mg) was mixed with 10 μl of 5 μM internal standards of d‐JA (2,4,4‐d3; acetyl‐2,2‐d2 JA [CDN Isotopes]), d6‐SA (Sigma‐Aldrich), and 500 μl phytohormone extraction buffer (1‐propanol/water/HCl [2:1:0.002 vol/vol/vol]). The samples were agitated for 30 min at 4 °C under darkness and then 500 μl dichloromethane was added to each sample. The samples were again agitated for 30 min at 4 °C in darkness and then centrifuged at 17,000 × g for 5 min. The lower organic layer of each sample was transferred to a glass vial for evaporation under nitrogen gas. Samples were resuspended in 150 μl methanol, transferred to a 1.5 ml microcentrifuge tube, and centrifuged at 17,000 × g for 2 min to separate any debris. Approximately 90 µl of supernatant of each sample was transferred into autosampler vials for LC‐MS/MS. The simultaneous detection of several phytohormones used methods of Müller and Munné‐Bosch (2011) with modifications. An Ascentis Express C‐18 Column (3 cm × 2.1 mm, 2.7 µm) (Sigma‐Aldrich) connected to an API 3200 LC‐MS/MS (Sciex) using electrospray ionization with multiple reaction mentoring was used. The injection volume was 10 μl and had a 600 μl/min mobile phase consisting of solution A (0.2% acetic acid in water) and solution B (0.2% acetic acid in acetonitrile) with a gradient consisting of 0.5 min–10% B, 1 min–20% B, 21 min–70% B, 24.6 min–100% B, 24.8 min–10% B, 29 min–stop. All hormones were quantified by comparing against isotopically labelled internal standards from Sigma‐Aldrich and Cayman Chemical.

## Data Availability

Research data are not shared.
