# Supplementary figures and images for: Green leaf volatiles and jasmonic acid enhance susceptibility to anthracnose diseases caused by Colletotrichum graminicola in maize
Source: Mol Plant Pathol. 2020 Feb 27;21(5):702–15. doi: 10.1111/mpp.12924 (PMC7170777; doi:10.1111/mpp.12924)

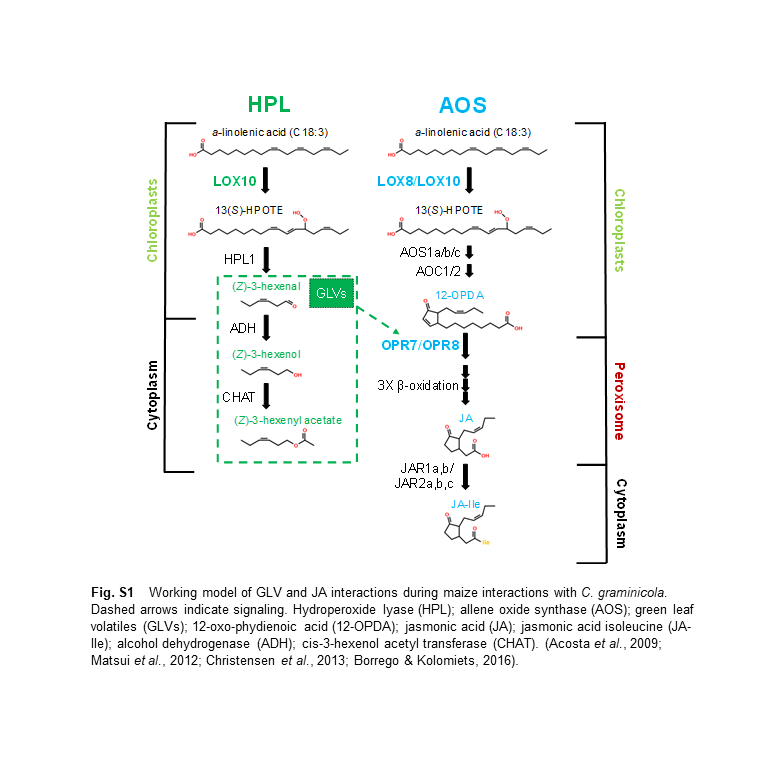

Supplement: Supplementary file 1 [file MPP-21-702-s001.tif]

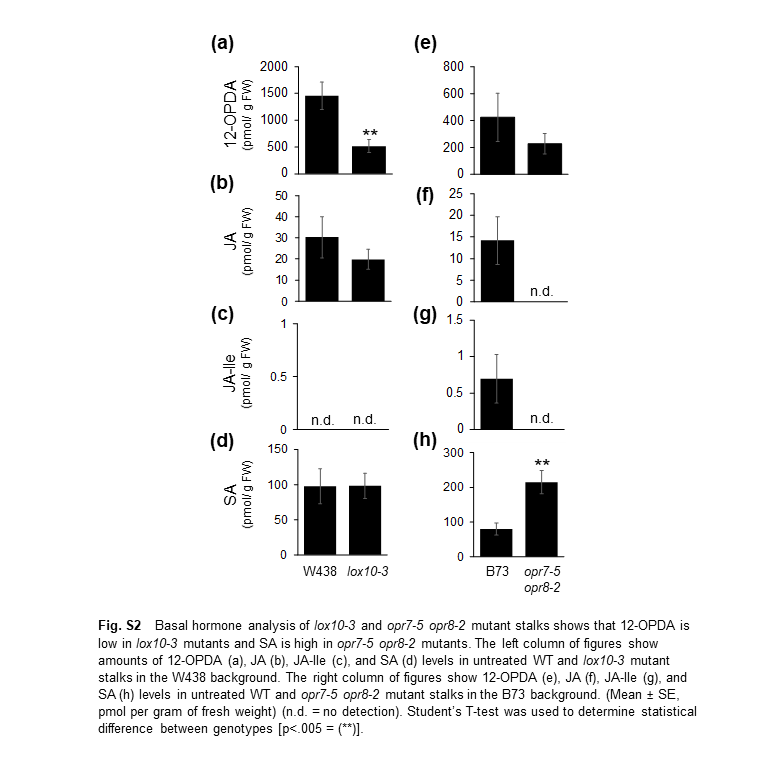

Supplement: Supplementary file 2 [file MPP-21-702-s002.tif]

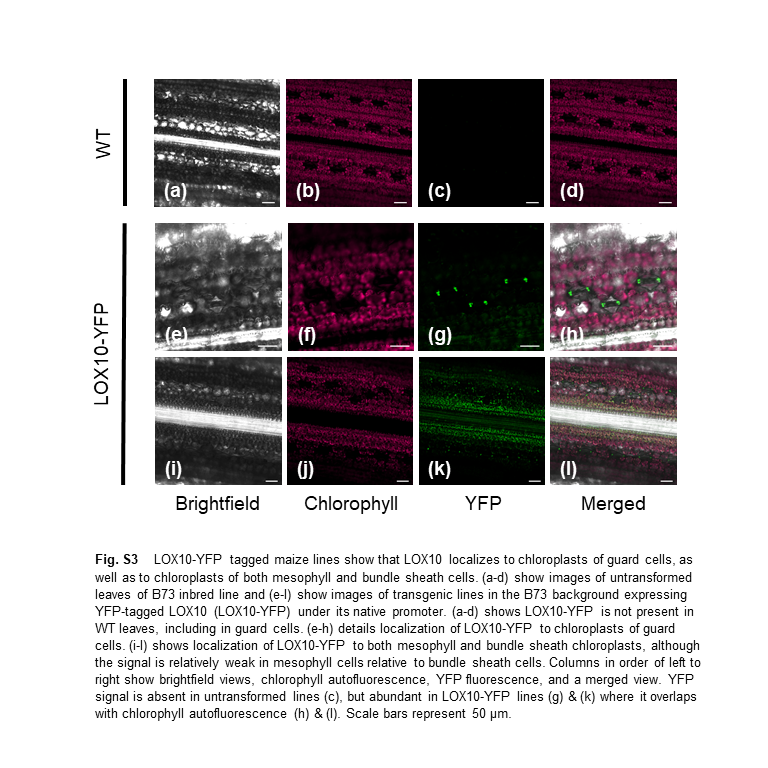

Supplement: Supplementary file 3 [file MPP-21-702-s003.tif]
